# Serum Cytokeratin-18 levels as a prognostic biomarker in advanced liver disease: a comprehensive meta-analysis

**DOI:** 10.1007/s10238-024-01423-y

**Published:** 2024-07-18

**Authors:** Xin Zhang, Jiangguo Li, Li Jiang, Yuexia Deng, Licheng Wei, Xing Li

**Affiliations:** 1 Department of Gastroenterology, The Fourth Hospital of Changsha, Changsha City, Hunan Province 410006 People’s Republic of China; 2 Department of Critical Care Medicine, Changsha Hospital of Traditional Chinese Medicine (Changsha No. 8 Hospital), 22 Xingsha Avenue, Changsha City, Hunan Province 410100 People’s Republic of China

**Keywords:** Advanced liver disease, Cytokeratin-18, Mortality, Liver transplantation, Meta-analysis

## Abstract

Cytokeratin-18 (CK-18) is a marker of hepatic cell death. Serum CK-18 could serve as a prognostic marker for patients with advanced liver disease (ALD). This meta-analysis aims to explore the association between total CK-18 (M65) and caspase-cleaved CK-18 (M30) levels with the prognosis of ALD patients. Relevant longitudinal observational studies were identified through comprehensive searches of the Medline, Web of Science, and Embase databases. A random-effects model was utilized to synthesize the findings, accommodating heterogeneity among studies. The analysis included 14 datasets from 11 studies. Elevated serum CK-18 levels at admission were linked to a higher risk of death or liver transplantation during follow-up. This association was consistent for both M65 (risk ratio [RR] 1.99, 95% confidence interval [CI] 1.65 to 2.40, *p* < 0.001; I^2^ = 43%) and M30 (RR 1.94, 95% CI 1.57 to 2.40, *p* < 0.001; I^2^ = 46%). Subgroup analysis revealed that the relationship between serum M65 levels and adverse outcomes was attenuated in studies using multivariate analysis compared to those using univariate analysis (RR 1.78 vs. 2.80, *p* for subgroup difference = 0.03). Further subgroup analyses indicated that the prognostic significance of CK-18 for ALD patients was not significantly influenced by study design, methods of determining CK-18 cutoff values, or follow-up durations. Elevated serum CK-18 levels at admission indicate a poor prognosis in patients with ALD. This finding holds for both M65 and M30.

## Introduction

Patients with advanced liver diseases (ALD), including conditions like progressed fibrosis, cirrhosis (both compensated and decompensated), and acute-on-chronic liver failure (ACLF), experience substantial impairment in liver function [1–3]. These patients often face complications such as ascites, infection, hepatic encephalopathy, and variceal bleeding [4], leading to an increased risk of mortality [5, 6]. The management of individuals with ALD requires a coordinated approach aimed at slowing down disease progression, managing associated complications, and considering liver transplantation as the ultimate treatment option for suitable candidates [7, 8]. Therefore, it is crucial to effectively assess the risk of patients with ALD.

Cytokeratin-18 (CK-18), an intermediate filament protein found mainly in epithelial cells, has become a possible indicator for predicting outcomes in liver diseases [9]. When hepatocyte apoptosis occurs, CK-18 cleaves, and its fragments are released into the bloodstream, making it a promising option for non-invasive prediction [10, 11]. At present, both serum levels of total CK-18 (M65) and caspase-cleaved CK-18 (M30) can be assessed in clinical settings [12]. Although some initial observational studies have suggested the potential prognostic importance of serum CK-18 levels in ALD patients [13–23], a comprehensive assessment through meta-analysis is still needed. Therefore, using a meta-analytical approach, this study examines the potential link between overall (M65) and caspase-cleaved CK-18 (M30) and the risk of death or liver transplantation in individuals with ALD. The composite outcome of the risk of death or liver transplantation was analyzed in this study primarily because these two endpoints represent the most significant and severe consequences of ALD, which comprehensively evaluated the prognosis of these patients.

## Methods

The current meta-analysis followed the protocols specified in the Preferred Reporting Items for Systematic Reviews and Meta-Analyses (PRISMA 2020) [24, 25] and the Cochrane Handbook for Systematic Reviews and Meta-analyses [26] throughout the stages of study design, data collection, statistical analysis, and interpretation of results.

### Literature search

To identify studies relevant to the aim of the meta-analysis, we searched Medline, Web of Science, and Embase utilizing comprehensive search terms: ("Cytokeratin-18" OR "Cytokeratin 18" OR "Keratin-18" OR "Keratin 18" OR "CK-18" OR "CK 18" OR "CYK18" OR "CYK-18" OR "KRT18" OR "KRT-18") AND ("cirrhosis" OR "cirrhotic" OR "liver fibrosis" OR "liver" OR "hepatic" OR "hepatitis" OR "liver failure") AND ("prognosis" OR "survival" OR "mortality" OR "death" OR "deaths" OR "transplant" OR "transplantation"). The search was restricted to human studies, specifically focusing on full-length articles published in peer-reviewed journals in the English language. Additionally, the references of relevant original and review articles were manually examined to identify potentially pertinent studies. The literature encompassing the period from the establishment of the databases to February 28, 2024, was thoroughly screened.

### Inclusion and exclusion criteria

The inclusion criteria for studies potentially eligible for this meta-analysis were as follows: (1) Observational studies with longitudinal follow-up, published as full-length articles, encompassing cohort studies, nested case–control studies, and post-hoc analyses of clinical trials; (2) Participants must be adults aged 18 years or older, diagnosed with ALD, including severe hepatitis, advanced fibrosis, cirrhosis (both compensated and decompensated), and acute-on-chronic liver failure (ACLF); (3) Serum levels of M65 and/or M30 must be measured at admission, using methodologies and cutoff values consistent with those in the original studies; and (4) Studies must report on the incidence of a composite outcome of all-cause death or liver transplantation in patients with higher versus lower serum CK-18 levels at baseline.

Exclusion criteria included: (1) Studies exclusively involving patients with hepatocellular carcinoma; (2) studies including patients post-liver transplantation; (3) studies that did not measure serum CK-18 levels or did not report the specified outcomes; and (4) preclinical studies, reviews, or editorials. In studies with overlapping populations, the one with the largest sample size was selected for inclusion in the meta-analysis.

### Study quality evaluation and data extraction

The literature search, study selection, quality assessment, and data extraction were independently conducted by two authors. Discrepancies were resolved through consultation with the corresponding author. The quality of included studies was evaluated using the Newcastle–Ottawa Scale (NOS) [27]), which considers three main dimensions: the selection of cases and controls, the comparability of groups, and the ascertainment of exposure. Data collected from each study included details such as the author, publication year, country, study design, participant demographics (diagnosis, sample size, age, sex, and mean Model for End-stage Liver Disease [MELD] score at baseline), serum CK-18 measurement methods, CK-18 variant (M65 or M30), criteria for CK-18 cutoff determination, average follow-up length, and adjusted variables in the analysis of the relationship between serum CK-18 levels and the occurrence of composite outcomes during follow-up.

### Statistical analysis

The relationship between serum CK-18 and the incidence of the composite outcome of death or liver transplantation in patients with ALD was assessed by calculating risk ratios (RRs) and corresponding 95% confidence intervals (CIs). RRs and standard errors (SEs) were determined using 95% CIs or p-values, with a subsequent logarithmical transformation applied to stabilize and normalize the variance. Study heterogeneity was evaluated using the Cochrane Q test and I^2^ statistics, with an I^2^ value greater than 50% indicating significant statistical heterogeneity [28]. A random-effects model was employed to combine the results, considering the influence of heterogeneity [26]. Sensitivity analyses were conducted by omitting one study at a time to further examine the findings. The study conducted predefined subgroup analyses to assess the impact of study characteristics on the outcome, such as study design, methods for determining the cutoff of serum CK-18, follow-up duration, and analytic model (univariate or multivariate). Funnel plots were constructed and visually inspected for symmetry to estimate publication bias in the meta-analysis [29].

Additionally, an Egger's regression test was conducted [29]. The statistical analysis was conducted using RevMan (Version 5.1; Cochrane Collaboration, Oxford, UK) and Stata software (version 12.0; Stata Corporation, College Station, TX). A two-sided *p* < 0.05 indicates statistical significance.

## Results

### Study inclusion

**Fig. 1:**
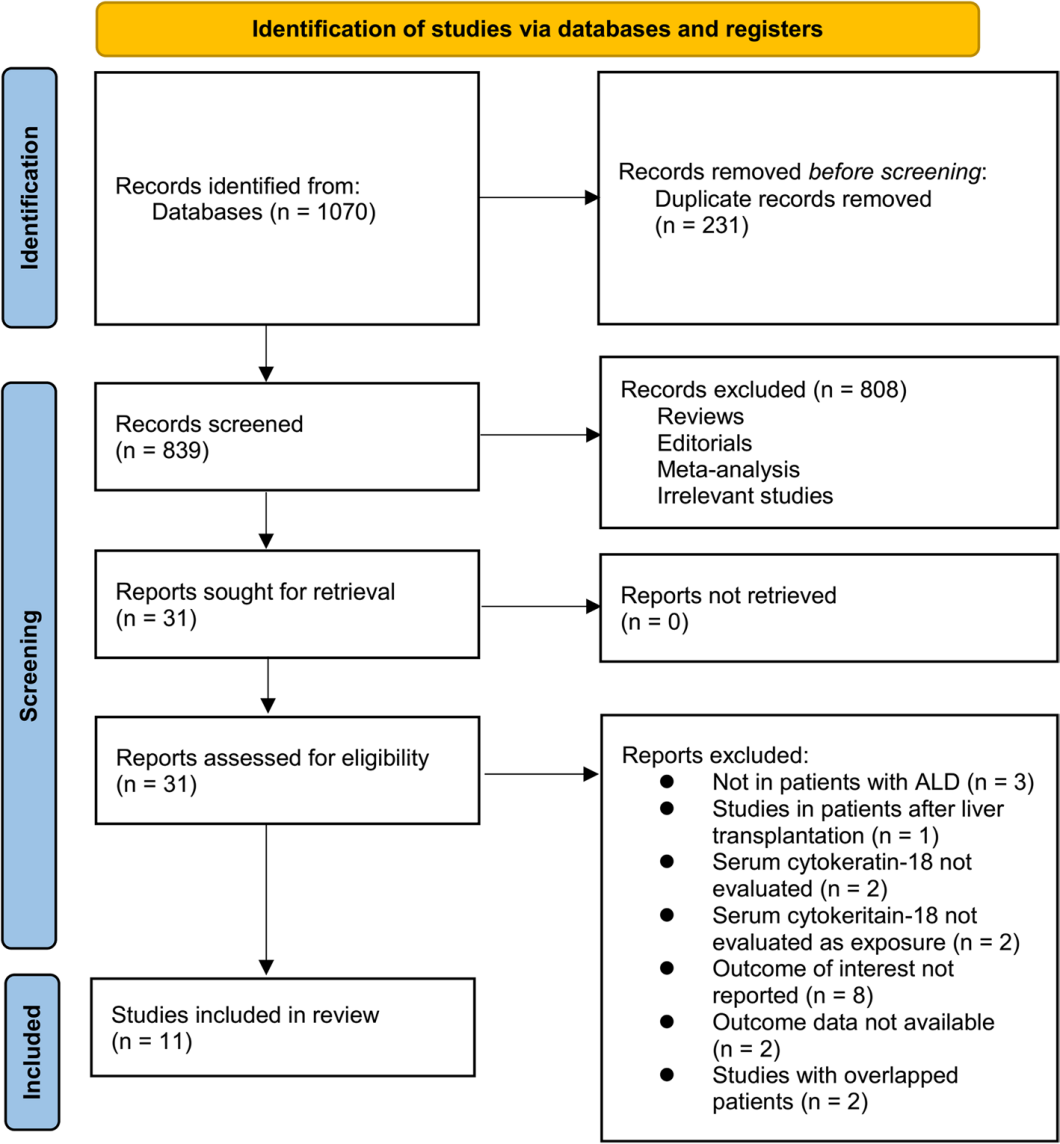
The flowchart depicts the process of database search and study inclusion

The study selection process is depicted in Fig. 1. Initially, a comprehensive search across three databases yielded 1070 potentially relevant records. Of these, 231 were excluded due to duplication. Further screening of titles and abstracts resulted in excluding 808 records, primarily because they did not align with the meta-analysis's objectives. The remaining 31 records underwent full-text review by two independent authors, excluding 20 studies for reasons detailed in Fig. 1. Consequently, 11 studies were deemed appropriate for the quantitative analysis [13–23].

### Overview of study characteristics

Table 1 presents the summarized characteristics of the included studies. Since one study included a test and a validation cohort [18], one study included patients with and without ACLF [17], and another included patients with and without active alcohol drinking [22], these datasets were included independently. Accordingly, 14 datasets from 11 studies involving 2944 adult patients with ALD were included [13–23], which involved patients of severe alcoholic hepatitis, cirrhosis, and ACLF. These studies were published between 2015 and 2023. All were prospective studies except two, retrospective cohort [21] and post-hoc analysis [20], respectively. The mean age of the patients was 42.8 to 61.0 years, and the mean MELD score at baseline was 9 to 28.1. Serum CK-18 was measured at admission with the enzyme-linked immunosorbent assay in all studies. All included studies measured M65, while in nine datasets, M30 was also measured [13, 15–17, 19–21, 23]. The cutoffs of a high serum CK-18 were determined via medians [13–15, 20, 23], tertiles [19], or Receiver Operating Characteristic curve analysis [13, 17, 18, 21, 22]. The mean follow-up duration was between 3 to 67.2 months. Univariate analyses were used in seven datasets when the association between serum CK-18 and prognosis of patients with ALD was investigated [16–18, 21, 23]. In the other seven datasets [13–15, 19, 20, 22], multivariate analyses were used, which adjusted potential confounding factors such as age, sex, and MELD score et al. to varying degrees. The NOS of the included studies were six to nine stars, suggesting overall moderate to good study quality (Table 2).

**Table 1:** Characteristics of the included studies

Study	Country	Design	Diagnosis	Patient number	Mean age (years)	Male (%)	Mean MELD score at admission	Methods for measuring serum CK-18	Type of CK-18 measured	Methods for CK-18 cutoff determination	Median follow-up duration (months)	Variables adjusted
Cao 2015	China	PC	HBV-ACLF	54	45.5	87	28.1	ELISA	M65 and M30	Median	3	Age, sex, TB, HBV-DNA, MELD score and CPS
Ding 2016	China	PC	HBV-ACLF	96	42.8	86.5	NR	ELISA	M65	Median	6	Age, sex, lactate, TB, Scr, INR, albumin, onset of ascites and encephalopathy
Waidmann 2016	Germany	PC	Cirrhosis (alcohol 52.1%, HBV 9.4%, HCV 26.1%)	211	56	64.5	15	ELISA	M65 and M30	Median	10.6	Age, sex, CRP, infection, MELD score and CPS
Mueller 2017	Germany	PC	Alcoholic cirrhosis	230	46.5	50.4	NR	ELISA	M65 and M30	ROC curve analysis derived	67.2	None
Payance 2018 test	France	PC	ACLD (alcohol 42%, HBV 7%, HCV 29%	139	56	77	13	ELISA	M65	ROC curve analysis derived	6	None
Payance 2018 validation	France	PC	ACLD (alcohol 18%, HBV 4%, HCV 75%	103	58	66	11	ELISA	M65	ROC curve analysis derived	6	None
Macdonald 2018 AD	Multiple countries	PC	Decompensated cirrhosis without ACLF	258	58	64	17	ELISA	M65 and M30	ROC curve analysis derived	3	None
Macdonald 2018 ACLF	Multiple countries	PC	Decompensated cirrhosis with ACLF	79	55	57	28	ELISA	M65 and M30	ROC curve analysis derived	3	None
Cao 2019	China	PC	HBV related decompensated cirrhosis	232	51	85.8	18.9	ELISA	M65 and M30	T3:T1	3	Age, sex, HBV-DNA, WBC, infection, presence of HE, and MELD-sodium
Atkinson 2020	UK	Post-hoc analysis	Severe alcoholic hepatitis	824	48.9	62.3	23	ELISA	M65 and M30	Median	3	Age, sex, MELD score, and treatments
Vatsalya 2020	USA	RC	Severe alcoholic hepatitis	84	47.4	64.3	NR	ELISA	M65 and M30	ROC curve analysis derived	3	None
Elkrief 2023 inactive	France	PC	Alcoholic cirrhosis	419	58	67	9	ELISA	M65	ROC curve analysis derived	24	Age, sex, MELD score, and FibroTest
Elkrief 2023 active	France	PC	Alcoholic cirrhosis	81	61	73	9	ELISA	M65	ROC curve analysis derived	24	Age, sex, MELD score, and FibroTest
Heinrich 2023	Germany	PC	Cirrhosis and hepatorenal syndrome (alcohol 54.1%, HBV 4.7%, HCV 16.8%)	134	58.5	57.5	21.2	ELISA	M65 and M30	Median	15	None

MELD, Model for end-stage liver disease; CK-18, cytokeratin-18; PC, prospective cohort; RC, retrospective cohort; HBV, hepatitis B virus; ACLF acute-on-chronic liver failure; HCV, hepatitis C virus; ACLD, advanced chronic liver disease; NR, not reported; ELISA, Enzyme-linked immunosorbent assay; ROC, Receiver operating characteristic; T, tertile; TB, total bilirubin; SCr, serum creatinine; INR, international normalized ratio; EBC, white blood cells; CPS, Child–Pugh Class; HE, hepatitis encephalopathy

**Table 2:** Study quality evaluation via the Newcastle–Ottawa scale

Study	Representativeness of the exposed cohort	Selection of the non-exposed cohort	Ascertainment of exposure	Outcome not present at baseline	Control for age and sex	Control for other confounding factors	Assessment of outcome	Enough long follow-up duration	Adequacy of follow-up of cohorts	Total
Cao 2015	1	1	1	1	1	1	1	1	1	9
Ding 2016	1	1	1	1	1	0	1	1	1	8
Waidmann 2016	1	1	1	1	1	1	1	1	1	9
Mueller 2017	1	1	1	1	0	0	1	1	1	7
Payance 2018 test	1	1	1	1	0	0	1	1	1	7
Payance 2018 validation	1	1	1	1	0	0	1	1	1	7
Macdonald 2018 AD	1	1	1	1	0	0	1	1	1	7
Macdonald 2018 ACLF	1	1	1	1	0	0	1	1	1	7
Cao 2019	1	1	1	1	1	1	1	1	1	9
Atkinson 2020	0	1	1	1	1	1	1	1	1	8
Vatsalya 2020	0	1	1	1	0	0	1	1	1	6
Elkrief 2023 inactive	1	1	1	1	1	1	1	1	1	9
Elkrief 2023 active	1	1	1	1	1	1	1	1	1	9
Heinrich 2023	1	1	1	1	0	0	1	1	1	7

### Serum level of overall CK-18 and prognosis of ALD

**Fig. 2:**
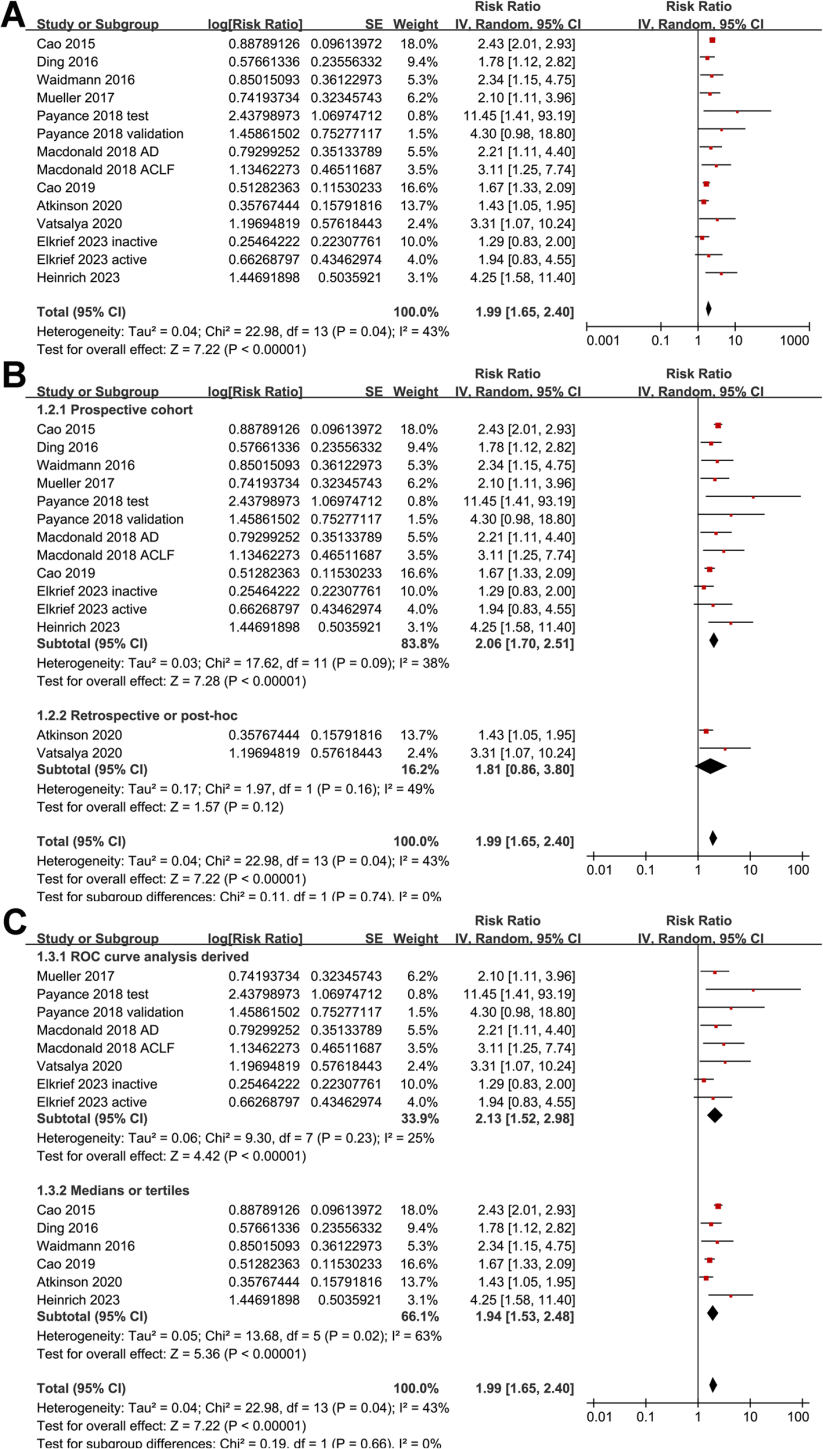
Forest plots for the meta-analysis of the association between serum level of overall CK-18 (M65) and the composite outcome of mortality or liver transplantation in patients with ALD; **A** forest plots for the overall meta-analysis; **B** forest plots for the subgroup analysis according to study design; and **C** forest plots for the subgroup analysis according to the methods for determining the cutoff of CK-18. The red square indicates the effect estimate (RR) of each included study, and the error bars extended on either side of the effect estimate represent the 95% CI for the effect estimate

**Fig. 3:**
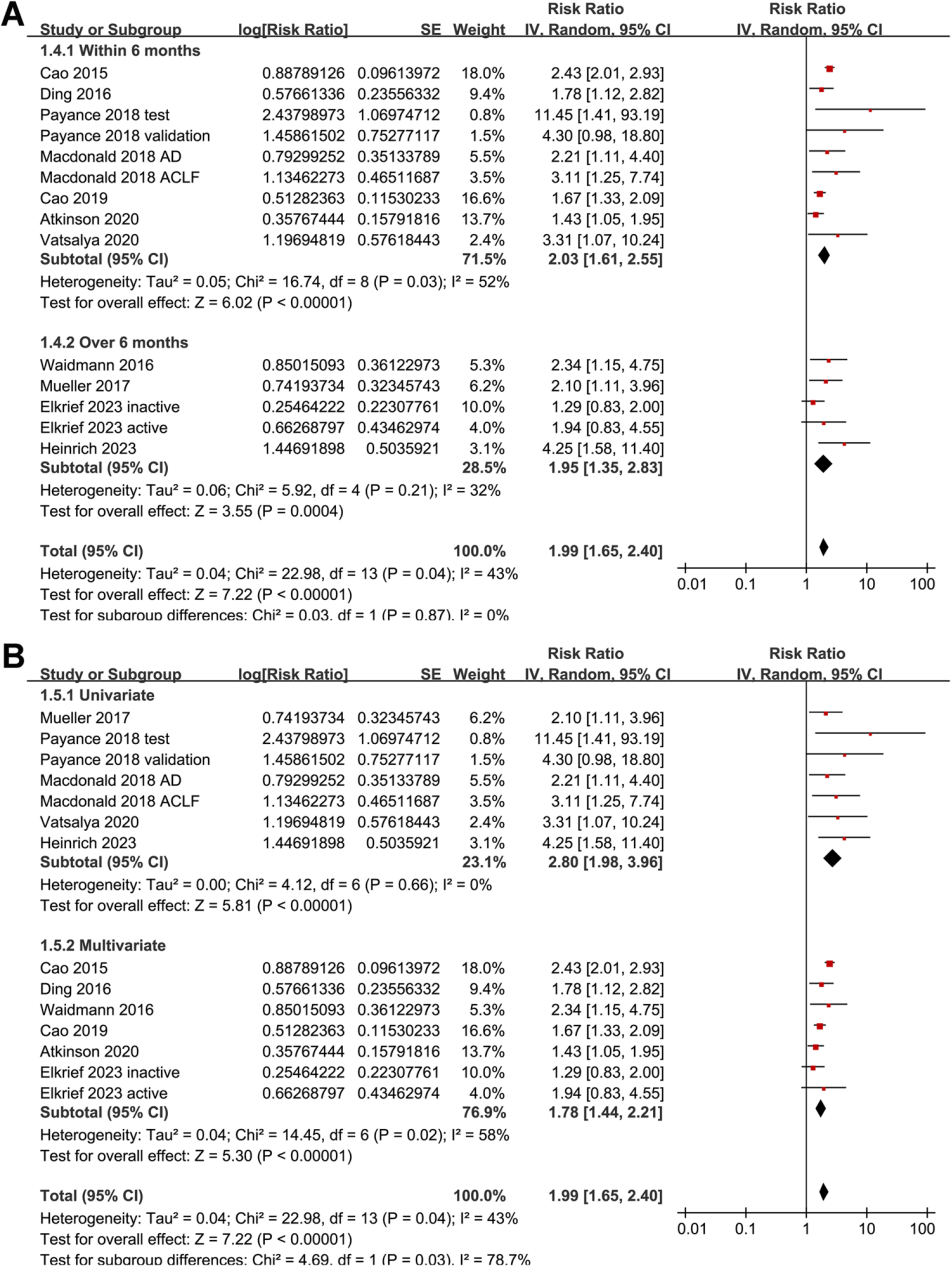
Forest plots for the subgroup analyses of the association between serum level of overall CK-18 (M65) and the composite outcome of mortality or liver transplantation in patients with ALD; **A** forest plots for the subgroup analysis according to follow-up durations; and **B** forest plots for the subgroup analysis according to analytic models. The red square indicates the effect estimate (RR) of each included study, and the error bars extended on either side of the effect estimate represent the 95% CI for the effect estimate

Pooled results of 14 datasets [13–23] with a random-effects model showed that compared to those with a lower M65, a higher serum M65 at admission was associated with an increased risk of death or liver transplantation during follow-up (RR 1.99, 95% CI 1.65 to 2.40, *p* < 0.001; I^2^ = 43%; Fig. 2A). Sensitivity analysis excluding one dataset at a time showed similar results (RR 1.85 to 2.08, *p* all < 0.05). Further subgroup analysis did not show that differences in study design (*p* for subgroup difference = 0.74; Fig. 2B), the methods for determining the cutoff of M65 (*p* for subgroup difference = 0.66; Fig. 2C), or the follow-up duration (*p* for subgroup difference = 0.87; Fig. 3A) could significantly affect the association. Interestingly, the association between serum M65 and the risk of death or liver transplantation was weakened in multivariate studies compared to univariate studies (RR 1.78 versus 2.80, *p* for subgroup difference = 0.03; Fig. 3B).

### Serum level of cleaved CK-18 and prognosis of ALD

**Fig. 4:**
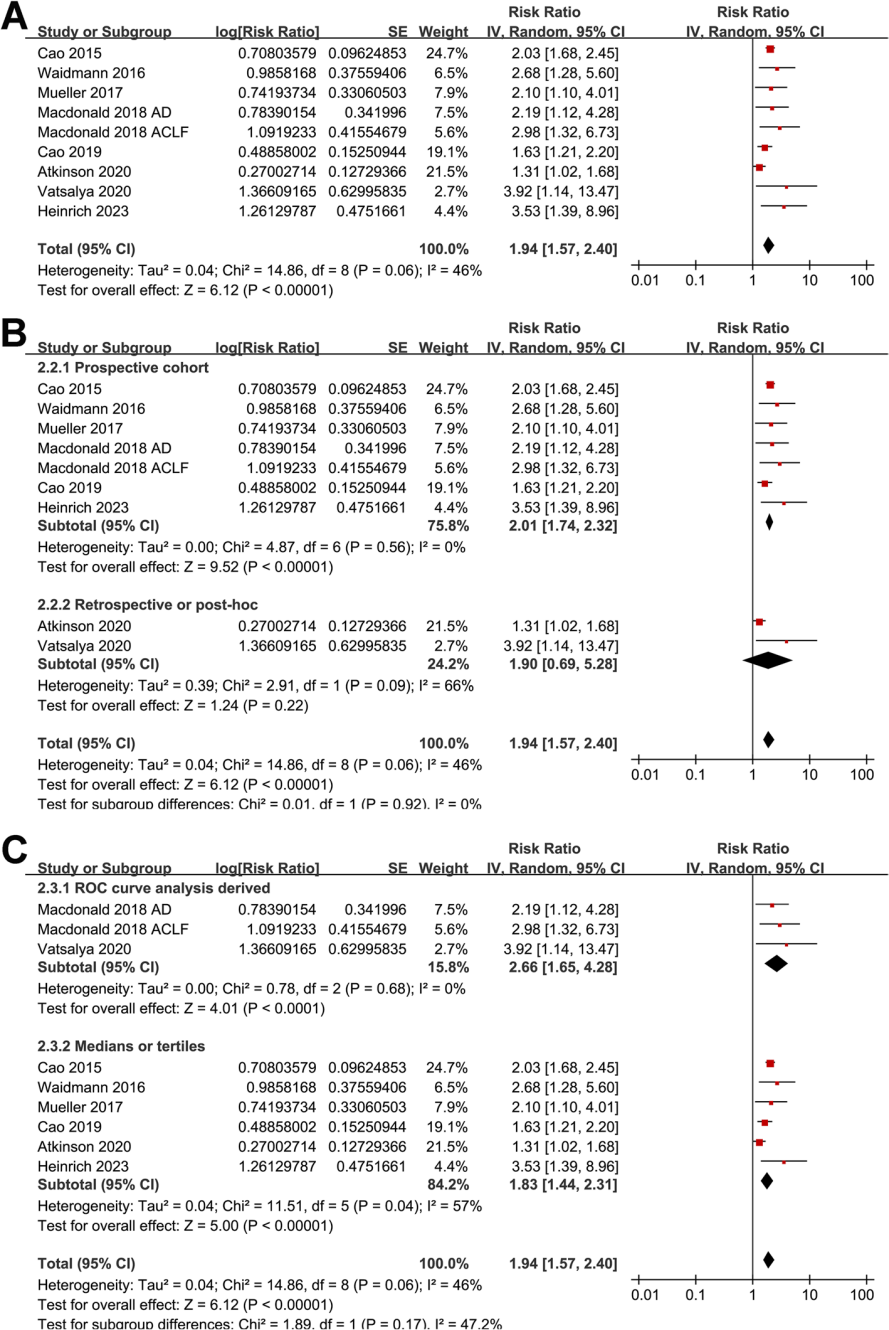
Forest plots for the meta-analysis of the association between serum level of cleaved CK-18 (M30) and the composite outcome of mortality or liver transplantation in patients with ALD; **A** forest plots for the overall meta-analysis; **B** forest plots for the subgroup analysis according to study design; and **C** forest plots for the subgroup analysis according to the methods for determining the cutoff of CK-18. The red square indicates the effect estimate (RR) of each included study, and the error bars extended on either side of the effect estimate represent the 95% CI for the effect estimate

**Fig. 5:**
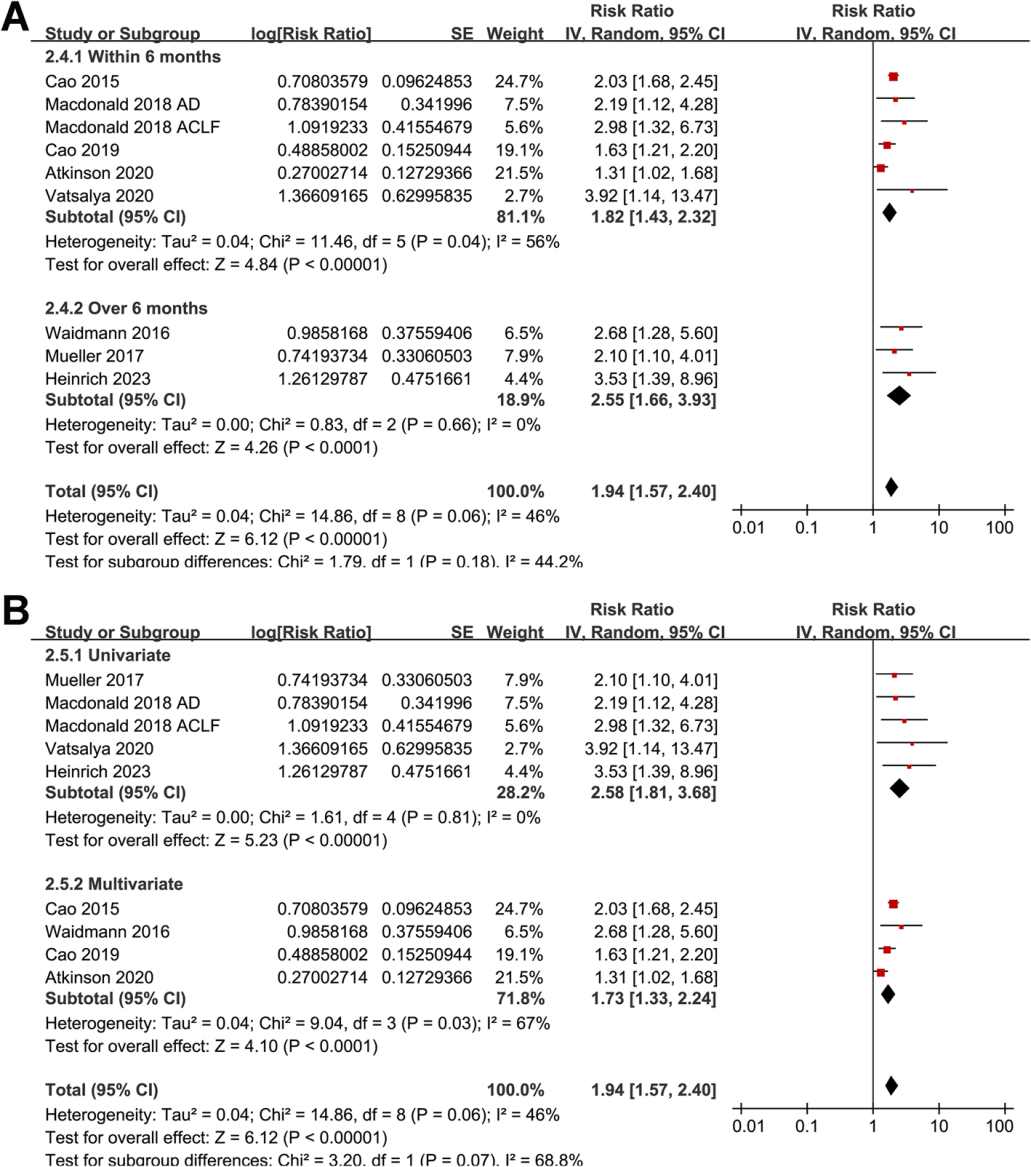
Forest plots for the subgroup analyses of the association between serum level of cleaved CK-18 (M30) and the composite outcome of mortality or liver transplantation in patients with ALD; **A** forest plots for the subgroup analysis according to follow-up durations; and **B** forest plots for the subgroup analysis according to analytic models. The red square indicates the effect estimate (RR) of each included study, and the error bars extended on either side of the effect estimate represent the 95% CI for the effect estimate

Pooled results of nine datasets [13, 15–17, 19–21, 23] showed that a higher M30 at admission was also associated with an increased risk of death or liver transplantation in patients with ALD during follow-up (RR 1.94, 95% CI 1.57 to 2.40, *p* < 0.001; I^2^ = 46%; Fig. 4A). Sensitivity analysis by omitting one dataset at a time did not significantly change the results (RR 1.89 to 2.07, *p* all < 0.05). Results of subgroup analyses showed that the association was not significantly affected by differences in study design (*p* for subgroup difference = 0.92; Fig. 4B), methods for determining the cutoff of M30 (*p* for subgroup difference = 0.17; Fig. 4C), follow-up duration (*p* for subgroup difference = 0.18; Fig. 5A), or the analytic models (*p* for subgroup difference = 0.07; Fig. 5B).

### Publication *bias* evaluation

**Fig. 6:**
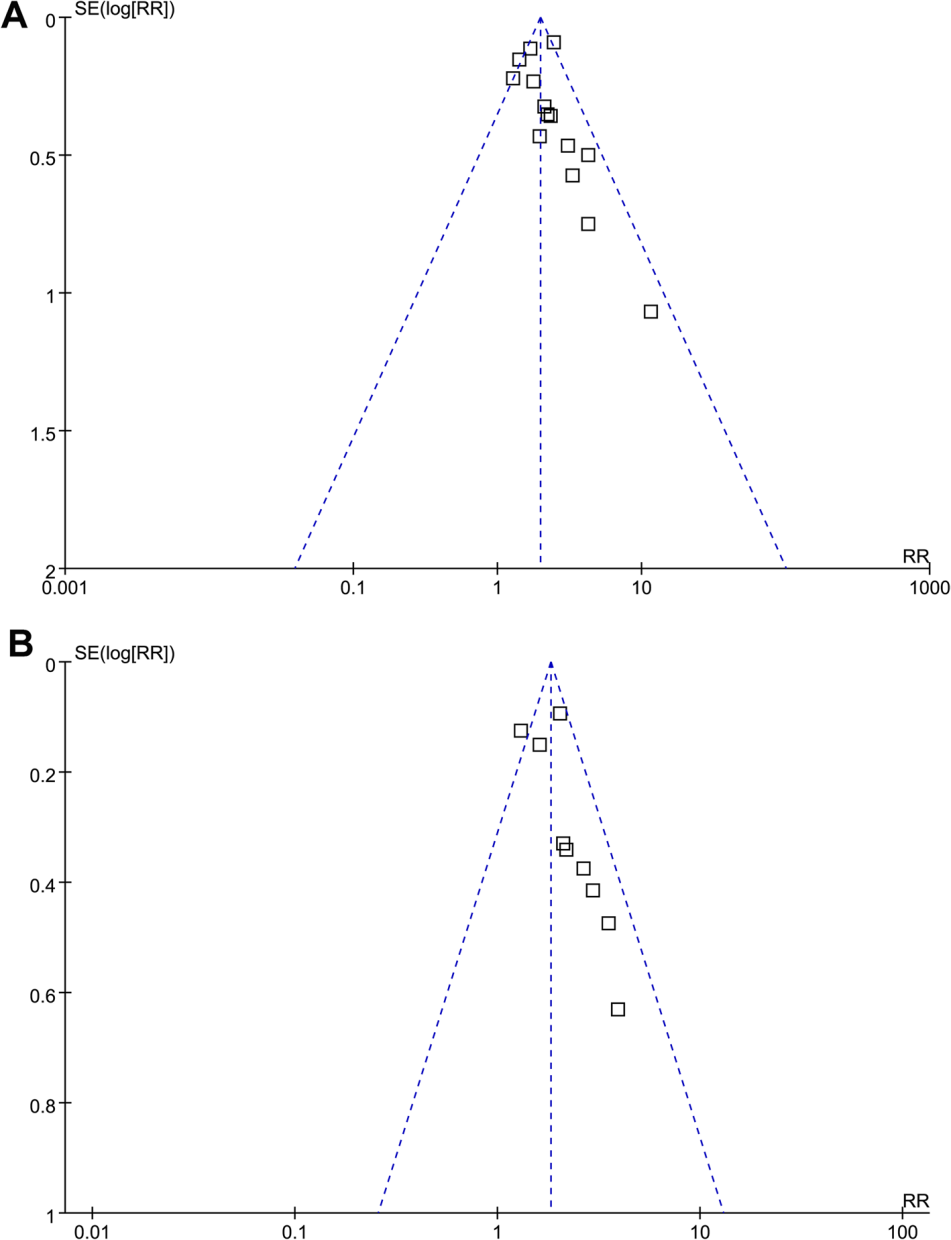
Funnel plots for the publication bias of the meta-analyses; **A** funnel plots for the meta-analysis of the association between serum level of overall CK-18 (M65) and the composite outcome of mortality or liver transplantation in patients with ALD; and **B** funnel plots for the meta-analysis of the association between serum level of cleaved CK-18 (M30) and the composite outcome of mortality or liver transplantation in patients with ALD

The symmetrical configuration of the funnel plots, as observed in the meta-analyses exploring the correlations between M65 and M30 and the composite outcome of mortality or liver transplantation in patients with ALD, indicates minimal risk of publication biases (Fig. 6A and B). Similarly, the outcomes of Egger's regression tests support the presence of low publication bias risks (*p* = 0.17 and 0.22, respectively).

## Discussion

This meta-analysis systematically assessed the association between serum CK-18 levels, specifically the overall (M65) and caspase-cleaved (M30) forms, and the prognosis of patients with ALD. Our results indicate that elevated serum CK-18 levels at admission may act as a prognostic indicator of poor outcomes for ALD patients, signified by an increased risk of death or liver transplantation during follow-up. Given the convenience and non-invasive nature of serum CK-18 measurement, our findings advocate for using serum CK-18 as a potential biomarker in the risk stratification of ALD patients.

To our knowledge, this is the inaugural meta-analysis that aggregates data on the relationship between serum CK-18 levels at admission and the subsequent risk of death or liver transplantation in ALD patients. Drawing on 14 datasets from 11 studies, the analysis uncovered a significant link between higher serum CK-18 levels and a negative prognosis in ALD patients. This correlation was consistently noted for both the M65 and M30 isoforms of CK-18.

The findings are in line with previous studies suggesting CK-18 as a marker of hepatic cell death [10], which could potentially signify the severity of liver injury and the progression of ALD. Subgroup analyses were conducted to explore potential sources of heterogeneity and further elucidate the observed associations' robustness. Interestingly, the subgroup analysis indicated that the association between serum M65 levels and the risk of death or liver transplantation was somewhat attenuated in multivariate studies compared to univariate studies. For the meta-analysis with M30, a trend of reduced association between serum M30 and the prognosis of ALD was also observed in multivariate studies compared to univariate studies (RR 1.73 versus 2.58, *p* = 0.07). This observation suggests that while serum CK-18 levels may independently predict outcomes in ALD patients, other clinical variables might influence the prognostic value when adjusting for multiple factors in multivariate analyses. Moreover, additional subgroup analyses were performed to assess the impact of the study design, methods for determining the cutoff of CK-18, and follow-up durations on the association between CK-18 levels and ALD prognosis. The results from these analyses revealed that the association between CK-18 and patient prognosis remained consistent across different study designs and methodologies, indicating the robustness and generalizability of the findings.

Pathophysiologically, CK-18 is an intermediate filament protein found in abundance within epithelial cells and provides structural support to the cells [30]. It is released during hepatocyte death through necrosis and apoptosis processes [31]. During necrosis, full-length CK-18 is passively released from dying cells, while during apoptosis, it is released in the form of cleaved CK-18 [31]. Monoclonal antibodies M65 and M30 can detect overall and cleaved forms of CK-18 in peripheral circulation, respectively [32, 33]. This detection reflects the severity of hepatic necrosis and apoptosis. Both forms of CK-18 have demonstrated better sensitivity for diagnosing liver damage when compared to alanine aminotransferase [34]. An early investigation found a connection between higher levels of serum M65 and M30 and the severity of liver fibrosis in individuals with alcoholic liver disease [35]. In non-alcoholic fatty liver disease patients, a meta-analysis indicated that elevated CK-18 levels might be beneficial for identifying non-alcoholic steatohepatitis and fibrosis, particularly for M65 [36]. For patients with chronic hepatitis B virus infection, it was suggested that M30 could serve as a promising non-invasive alternative to liver biopsy for predicting significant histological damage [37]. Additionally, in individuals with compensated alcohol-associated liver disease, increased levels of M30 were found to be useful in detecting severe hepatic inflammatory activity and predicting the occurrence of liver-related events such as acute decompensation [38]. These findings may partially explain the link between elevated serum CK-18 and an increased risk of death or need for liver transplantation in patients with ALD.

The strengths of this meta-analysis lie in its extensive search strategy, strict inclusion criteria, utilization of a random-effects model to manage heterogeneity across studies, and the performance of multiple sensitivity and subgroup analyses to ascertain the reliability of the findings. However, it is essential to recognize several limitations. First, the variability in patient populations, CK-18 cutoff values, follow-up lengths, and adjustments for potential confounding factors among the included studies might have introduced heterogeneity and potential biases. Through subgroup analyses, attempts were made to assess the impact of specific study characteristics on the meta-analysis outcomes. However, caution is advised in interpreting these subgroup analyses due to the limited datasets and reliance on study-level rather than individual patient-level data.

Consequently, there is a compelling need for large-scale prospective studies to verify our results and further investigate the influence of these study characteristics. Even though subgroup analyses confined to studies incorporating multivariate analyses yielded similar results, the potential existence of unadjusted factors that could affect the relationship between CK-18 and the incidence of mortality or liver transplantation in ALD patients, such as the severity of systemic inflammation [39]. Additionally, we are focused and cannot be disregarded. Moreover, our focus was on the serum CK-18 level at admission. Observing dynamic changes in serum CK-18 following treatment in ALD patients and their potential correlations with clinical outcomes warrants future investigation. Lastly, this study was limited to observational studies, precluding the determination of a causal link between elevated serum CK-18 levels and the adverse prognosis of ALD patients.

## Conclusions

In conclusion, this meta-analysis reveals that elevated serum CK-18 levels at admission could be a prognostic marker for poor outcomes in patients with ALD. These results highlight the potential of CK-18 as a valuable biomarker for risk stratification and the clinical management of ALD patients. Nonetheless, further prospective studies employing standardized methodologies and larger cohorts are essential to corroborate these findings and uncover the mechanisms through which CK-18 levels influence the prognosis of ALD.

## Data Availability

The authors confirm that the data supporting the findings of this study are available within the article. Further inquiries can be directed to the corresponding author.

## Abbreviations

ACLF: Acute-on-chronic liver failure;
ALD: Advanced liver diseases
CK-1: Cytokeratin-18
MELD: Model for end-stage liver disease
NOS: Newcastle–Ottawa scale
RR: Risk ratios
SE: Standard error
